# The Treatment of Metastatic Non-Small Cell Lung Cancer in a New Era of Personalized Medicine

**DOI:** 10.3389/fonc.2015.00020

**Published:** 2015-02-03

**Authors:** Vera Hirsh

**Affiliations:** ^1^Department of Medical Oncology, McGill University Health Centre, Montreal, QC, Canada

**Keywords:** NSCLC, personalized medicine, lung cancer, NSCLC treatment, supportive care

Lung cancer is the leading cause of cancer-related mortality in Canada (1) and around the world. Non-small cell lung cancer (NSCLC) is the most frequent form of lung cancer, accounting for approximately 87% of cases and the majority of these are metastatic at the time of presentation (2, 3).

We have reached a plateau (4, 5) with different systemic chemotherapies, specifically platinum-based, which have been used to treat metastatic NSCLC for several decades; median survival improved to 8–10 months (from 4-6 months without treatment). Significant toxicities limited the number of cycles that could be administered (6).

Current recommendations for first-line treatment of advanced NSCLC use both histologic and molecular diagnostics in designing the course of treatment (7, 8). We have learned the importance of distinguishing between squamous and non-squamous histologies (9) in order to choose an appropriate chemotherapy regimen. The algorithms for first-line treatment of advanced NSCLC recommend using both histologic and molecular diagnostics in designing the treatment (7, 10, 11). This in turn requires an adequate amount of biopsied tumor tissue in order to be able to perform all the necessary testing, which is needed for right decisions (12). Tumor aspirations for the diagnosis are not acceptable anymore.

Recent advances in understanding signaling pathways for malignant cells, interconnections in those pathways, the importance of various receptors (13–15), and biomarkers, and also the interplay between various oncogenes have led to the development of targeted treatments that are improving not only the efficacy of the treatments, but also safety benefits, less toxicity (16) with improvement of patient’s quality of life (17) in this palliative setting.

These treatments are aimed at specific (especially genetic) alterations in the malignant cells. Various NSCLC subtypes are associated with potentially targetable biomarkers such as mutation of the epidermal growth factor receptors (EGFR) (18–22), KRAS (23), or the presence of echinoderm microtubule-associated protein-like 4 (EML-4) and anaplastic lymphoma kinase (ALK) fusion genes, ALK rearrangements (13, 15). C-Met over-expression or amplification (24–27), are playing a role in the development of resistance to the therapies (28), i.e., with EGFR-TKIs. T790M mutation on Exon 20 in the EGFR domain is the most frequent cause of the development of this resistance (29).

Knowledge about the advantages of treatments with targeted agents in advanced NSCLC is rapidly growing, but the hope is to eventually apply this knowledge to earlier stages of NSCLC and thus to increase the cure rate of these patients. Combining various targeted agents or sequencing them properly will be of the utmost importance in the new era of personalized targeted therapy (30). Many clinical trials are ongoing to help us make the appropriate decisions how to optimally treat advanced NSCLC in future (31, 32). Immunotherapy of advanced NSCLC (33) is one of the exciting areas of research and results of phase III trials are eagerly awaited.

Contributors in this issue of Frontiers in Thoracic Oncology describe the importance of team work (34) from diagnosis through various treatments to supportive care. They explain and emphasize the importance of the treatments of brain metastases (35) and bone metastases with new bone targeted agents (36). Management of adverse events when the new targeted agents are used (16) and analysis of patients’ health-related quality of life (HR QOL) (17) and the impact on patients’ performance status (PS) are also discussed in this issue. It is very important to preserve a good PS of patients in order to make it possible for them to receive multiple lines of the treatments now available for advanced NSCLC.

Our review will cover the description starting with the interventional procedures (12), to treatments delivered by radiation oncologists (37), medical oncologists (10, 11, 34), including descriptions of ongoing trials to provide a glimpse of the future (31, 32). The importance of early supportive care (38), which should be an integral part of active care from the start of treatment of advanced NSCLC, will also be discussed.

We hope to provide a complete review of present and future approaches to personalized medicine in advanced NSCLC, reflecting the present views, and practices in Canada.

## Conflict of Interest Statement

The author declares that the research was conducted in the absence of any commercial or financial relationships that could be construed as a potential conflict of interest.
